# The phenomenology and impact of hallucinations concerning the deceased

**DOI:** 10.1192/bjo.2021.960

**Published:** 2021-08-16

**Authors:** Evelyn Elsaesser, Chris A. Roe, Callum E. Cooper, David Lorimer

**Affiliations:** Chavannes de Bogis, Switzerland; Centre for Psychology & Social Sciences, University of Northampton, UK; University of Northampton, UK; Scientific and Medical Network, UK

**Keywords:** Phenomenology, qualitative research, transcultural psychiatry, anomalous experiences, childhood experience

## Abstract

**Background:**

People who have suffered the loss of a loved one may subsequently report sensory experiences of the deceased (termed ‘after-death communications’, or ADCs). Such encounters are common and can be a source of comfort to the bereaved. Nevertheless, there has been limited empirical investigation of this phenomenon, and consequently mental health professionals feel ill-equipped to support those who disclose them.

**Aims:**

To map the phenomenology of ADCs, and identify covariates and effects upon the recipient.

**Method:**

We conducted an online mixed-methods survey comprising 194 items about all aspects of ADCs. A purposive sample of 1004 respondents across three language groups (English, French and Spanish) completed the survey.

**Results:**

The most common form of ADC was during sleep, but large numbers of cases involved sensory modalities of touch, sight, hearing, smell and sense of presence that externalised the phenomenon for the recipient. Variations in incidence with participant gender and language group suggest a psychosocial component. ADCs were typically regarded by the participant as deeply meaningful and comforting. Respondents reported significant increases in their sense of spirituality, but not religiosity.

**Conclusions:**

ADCs are a common feature of bereavement that occur unexpectedly, and are independent of any underlying pathology or psychological need. For the person experiencing the hallucination, they are important and meaningful events that they interpret in terms of continuing bonds with the deceased. This adaptive outcome may be stymied where mental health professionals trivialise or pathologise disclosures about ADCs.

It is 50 years since Rees published an account of interviews he had conducted with 227 widows and 66 widowers who were registered with his general practice.^1^ He was initially interested to identify factors that might be beneficial or obstructive to the bereavement process, given the observation that the death of a spouse frequently precipitated the death of the surviving widow or widower.^2^ During the course of his interviews, however, he was surprised to discover that almost half the people he spoke to disclosed that they had experienced hallucinations of their dead spouse (the term ‘hallucination’ is used here to refer to a sensory perception experienced in the absence of an external stimulus; it is intended to be ontologically neutral, and does not imply that such experiences are necessarily a consequence of disease or dysfunction). These often occurred over many years, and at the time of the interviews 106 people (36.1% of the sample) were still having them. The form of encounter varied, most commonly taking the form of a ‘sense of presence’ of the deceased (reported in 39.2% of cases), but also including visual (14.0%), auditory (13.3%) and tactile (2.7%) experiences. A majority of those reporting encounters with their deceased spouse regarded them as helpful in their recovery from loss, and Rees concluded that these hallucinations were normal and beneficial accompaniments of widowhood. Other researchers have been able to confirm this observation.^3–9^ These studies are remarkably consistent in finding that sense of presence experiences are most common, being reported by 40–60% of respondents; for experiences within a particular sensory modality there is less agreement, although auditory and visual experiences are typically more common than other sensory forms.^10^ The phenomenon has been given a variety of labels, including ‘post-bereavement hallucination’, ‘encounters with the dead’ and ‘after-death communications’ (ADCs),^11^ and these terms are used interchangeably here.

Representative sample surveys in a number of countries have also demonstrated that ADCs are quite common, even among those who are not recently bereaved. For example, Haraldsson^12^ reported on an Icelandic survey in which 31% reported that they had ‘perceived or felt the nearness of a deceased person’ (36% of women, 24% of men), and in the USA McCready and Greeley^13^ found that 27% of respondents answered affirmatively the question ‘Have you ever felt that you were really in touch with someone who had died?’ In the UK, an Ipsos MORI poll^14^ found that 17% of their sample claimed to have personally experienced a ‘ghost’, and a subsequent poll found that 10.4% had experienced an ADC.^15^ In Germany, the incidence of having experienced an ‘apparition’ (described as perceiving something they took for a ‘ghost’ of someone who had died) was 15.8% (18.6% of women, 11.3% of men).^16^ These experiences seem to be independent of culture or religious affiliation.^17^

## ADCs and mental health

Despite their frequency of occurrence, there has been only limited academic interest in perceptions of the deceased during bereavement (Keen et al's review^10^ identified just 36 qualifying studies), possibly reflecting concerns about seeming to endorse claims that are incompatible with mainstream conceptions of reality^18^ – perhaps a legacy of Freud's description of sensing the presence of the deceased as ‘hallucinatory wishful psychosis’.^19^ Indeed, beliefs among the general public regarding the incidence, causes and consequences of encounters with the deceased reflect concerns that they are a consequence of psychological weakness or even pathology. For example, in Rees's original study, interviewees reported that they were reticent to share the experience with others – only 27.7% had mentioned it to anyone at all – citing fear of ridicule, or concern that the phenomenon was too personal or potentially upsetting to disclose to others. This is disappointing, given that the high frequency of such hallucinations among an otherwise non-clinical population challenges the notion that they are pathological *per se* (echoing a more general clinical debate^9,20–23^). Researchers who have specialised in the study of anomalous experiences have similarly concluded that they are not indicative of pathology.^15,24–26^ Hayes and Leudar^27^ have argued that the effect of ADCs depends on how recipients contextualise them to make them intelligible and meaningful, and that negative or challenging experiences may simply reflect negative or challenging aspects of their relationship with the deceased pre-mortem. Much of the literature invokes a continuing bonds model of bereavement^28^ that emphasises the importance of maintaining an emotional connection with the deceased as a normal and adaptive aspect of recovery from loss. From this perspective, post-mortem encounters can be beneficial; for example, in affording an opportunity (at least symbolically) to help make sense of the death and resolve the trauma arising from it, to settle unfinished business and say goodbye, as well as provide emotional or practical support for current difficulties. The contact is typically interpreted by the recipient as conveying (explicitly or implicitly) one or more of the following sentiments (which we have termed the ‘four Rs’): ‘reassuring’**,** I'm fine, don't worry about me, the troubles I had at the end of life are now behind me; ‘resolving’**,** settling old conflicts, allowing space for apologies and providing closure; ‘reaffirming’**,** continuing bond, affectionate, I love you, I will always be by your side, we'll meet again one day; and ‘releasing’**,** don't be sad, pursue your life, don't hold me back by your suffering.

Consequently, ADCs have been associated with reduced susceptibility to adverse consequences of bereavement such as loneliness, sleep problems, loss of appetite and weight loss.^27,29^ The greatest benefits are reported by those who are able to conceptualise and integrate the experiences, typically by drawing on spiritual or religious frameworks.^30^ Ironically, attempts at meaning making may be stymied by the reticence of mental health professionals to engage with ADCs in a therapeutic setting. Where clients have sought bereavement support, they have typically found therapists incapable or unwilling to provide a safe space for them to reflect on and make sense of anomalous experiences involving the deceased, and they have learned not to disclose them.^31–33^ Equally, therapists feel ill-prepared to deal with ADCs, citing the paucity of balanced, evidence-based information about the nature and incidence of such experiences, and unfounded concerns about associations with pathology (in the absence of comorbidity factors).^18,25,34^

## Aims

The present study represented an attempt to map in more detail and with a much larger sample the phenomenology of ADCs, their covariates and effects on the recipient, particularly in relation to recovery from the loss of a loved one. We sought to identify cultural aspects of the experience by testing whether differences existed in the nature and effects of ADCs across three language groups.

## Method

An extensive 194-item questionnaire was constructed, the main foci of which included an initial description in the respondent's own words of the ADC, to ensure their account was not biased by the specific questions we subsequently asked. This item generated over 150 000 words in response, and qualitative analysis of these data is reported elsewhere.^35^ Subsequently, respondents answered specific questions about the circumstances of occurrence, features of the experience as they related to each modality separately (including during sleep), the subject of the experience and details of their passing, impact on personal beliefs and implications for the grieving process. The questionnaire comprised closed questions with fixed response options (e.g. ‘Did you feel a physical contact allegedly initiated by the deceased? Yes, No, Unsure’) and open questions following affirmative responses that allowed for elaboration (e.g. ‘In what part of your body did you feel the contact and how did it occur?’). A copy of the questionnaire developed for this study is available as Supplementary File 1 available at https://doi.org/10.1192/bjo.2021.960, and can be found online at https://pure.northampton.ac.uk/ws/portalfiles/portal/25092859/Elsaesser_ADC_Questionnaire.pdf.

The hard-copy questionnaire was transposed into an online version, using the Online Surveys platform (https://www.jisc.ac.uk/online-surveys). Online delivery was preferred because this enabled greater outreach to participants, who could be provided with a web link and allowed to complete the survey at their convenience. Additionally, it enabled the questionnaire to be designed so that it responded ‘intelligently’; for example, if a respondent indicated that they had not had an auditory component to their experience, they would not be asked any follow-up questions that related only to auditory experiences. Versions of the survey were produced in three languages (English, French and Spanish), with content reviewed by native speakers.

The research project received ethical approval from the University of Northampton (approval number FHSRECSS00084) and was preregistered (reference: KPU Registry 1046) with the Koestler Unit Study registry at the University of Edinburgh (https://koestlerunit.wordpress.com/study-registry/registered-studies/). The survey landing page reminded participants of the nature of the study and of what participation would entail, including that data were volunteered anonymously, so it was not possible to withdraw data once submitted. Respondents confirmed that they consented to participate in order to progress to the questionnaire.

Participants were recruited with a purposive snowball sampling method by advertising the survey during public talks and through social media forums that specialise in ADCs and related phenomena. Interested parties were referred to the principal investigator's web page that gave further information about the project and provided a link to the survey. Each survey version was made ‘live’ for prespecified periods of time: the English version from 9 August 2018 to 31 January 2019 for the English version, from 15 September 2018 to 31 March 2019 for the French version and from 21 October 2018 to 30 April 2019 for the Spanish version.

Participants completed the survey in their own time. There was no facility to save progress so the whole questionnaire needed to be completed in a single session (although the web link would not time out and so could be left open for as long as required). Participants needed to complete the whole measure for their data to be submitted for subsequent analysis.

## Results

A total of 1004 completed responses were received. Initial screening removed 13 incomplete or spoiled submissions, leaving 991 viable cases (412 English, 434 French and 145 Spanish) from 143 male and 842 female participants (mean age 51.1 years, range 18–89 years). The finding that women are much more likely than men to report a hallucination involving the deceased (84.9% *v*. 14.5%, with four responding ‘other’ and two declining to answer this item) is typical of surveys of anomalous experience (e.g. Castro et al^15^), and is broadly comparable with Rees's sample (75.9% *v*. 24.1%). The largest demographic group (48.2%) were educated to university level, and a majority (79.2%) were in employment or retired. Among this sample, 86.4% reported that they were in good health, with 17.5% disclosing that they were depressed and 4.2% currently taking medication such as antidepressants (respondents could check more than one option so these figures need not add up to 100%). In Rees's sample the incidence of depression was similar for the hallucinated and non-hallucinated groups (17.5% and 18.0%, respectively), so that this does not seem to be a disposing factor. Indeed, 27% of our respondents had never been in mourning for the perceived deceased person or were not mourning anymore.

The forms of ADC experienced are summarised in Table 1 (respondents could select more than one modality); for comparison, we reproduce Rees's findings from interviewees who disclosed an ADC to him.

**Table 1:** Incidence of after-death communications by modality

	Male	Female	Overall	Rees (1975)^2^
Sleep	75 (52.1%)	538 (64.4%)	613 (62.4%)	–
Touch	59 (41.0%)	411 (49.4%)	472 (47.8%)	8 (5.8%)
Sight	74 (51.4%)	382 (45.5%)	459 (46.4%)	41 (29.9%)
Hearing	70 (48.6%)	358 (42.8%)	428 (43.4%)	39 (28.5%)
Sense of presence	49 (34.0%)	290 (34.6%)	339 (34.3%)	115 (83.9%)
Smell	33 (23.2%)	238 (28.5%)	271 (27.6%)	–

In the current study, experiences most commonly occurred during sleep. These were discounted by Rees as mere dreams and were excluded from Keen et al's summary review^5^ of post-mortem encounter surveys. However, when we asked respondents whether the experience was merely a dream, 36.6% asserted that it was not, and follow-up accounts suggest that respondents regarded them as different in quality to an ordinary dream and attributed an external agency to them. Those who had had dreams of deceased loved ones and an ADC during sleep made a clear distinction between the two types of experiences. It would have been interesting to discover whether ADCs are associated with particular stages of sleep; although the accounts we collected suggest that ADCs tend to occur during hypnagogic and hypnopompic periods as well as during dreams, unfortunately the questionnaire does not allow any finer distinctions to be made.

For Rees, by far the most common experience was a sense of presence, whereas for the current sample, this was relatively uncommon (albeit still reported by a third of respondents). This difference may be a function of the method of data collection: Rees conducted an exhaustive survey of qualifying persons in his practice, interviewing each person individually so that even subjectively trivial experiences may have been disclosed, whereas our participants had to be sufficiently motivated to seek out and complete an extensive survey, and some may have been reticent to share experiences that they perceived as less objectively impressive or evidential (such as vaguer ‘sense of presence’ cases), with the result that they are underrepresented here. Additionally, 79.8% of our respondents had several ADCs but were asked to answer questions in relation to the most significant one so as to avoid conflation, and so may have tended to choose their most ‘spectacular’ experience. In previous work, auditory and visual experiences are typically more common than other sensory forms,^17^ and these are also relatively common here; however, tactile experiences are notably more frequently reported here than for other surveys.^10^ We consider the phenomenology of these experiences in a separate paper.^35^

Women were significantly more likely than men to report an ADC occurring during sleep (*χ*^2^ = 8.05, *P* = 0.018), but none of the other modalities showed evidence of sex differences. We also considered whether there were cultural differences in ADC type that might be captured indirectly by comparing responses from the three language groups. These data are given in Table 2. Although there are no differences by language group in the incidence of cases involving touch or smell, there were significant differences for cases involving ADCs during sleep, sight, hearing and sense of presence, suggestive of a cultural component to how ADCs are experienced.

**Table 2:** After-death communications type by language group

	English	French	Spanish	*χ*^2^	*P-*value
Sleep	261 (63.3%)	249 (58.0%)	105 (72.4%)	12.894	0.012
Touch	190 (46.1%)	215 (50.5%)	67 (46.5%)	2.280	0.684
Sight	192 (46.5%)	179 (41.5%)	88 (60.7%)	19.266	0.0007
Hearing	198 (48.1%)	160 (37.3%)	72 (49.7%)	13.174	0.010
Sense of presence	103 (24.9%)	172 (40.2%)	64 (43.8%)	28.742	0.00001
Smell	107 (26.0%)	125 (29.5%)	42 (28.8%)	7.709	0.103

Although 54.7% of ADCs occurred in the evening or at night when lower lighting and vigilance levels might contribute to misperceptions, 42.0% occurred in the daytime; 30.6% were witnessed in daylight, with a further 14.7% experienced by electric light. For comparison, Haraldsson^17^ found that over half of his cases occurred either in daylight or full electric light, with a further 33% in twilight and only 10% in darkness. Surprisingly, 36.4% of our respondents reported that they were not alone at the time of their ADC, and of these, 21.0% asserted that the ADC was witnessed by their companions. Also related to the perceived evidentiality of the experiences, 24.4% of respondents stated that they had received information that was previously unknown to them (often concerning circumstances of the deceased's passing). These cases have a potential bearing on the ontological status of ADCs, as explored by a number of authors,^5,36,37^ and will be explored in a separate analysis.

ADCs were typically regarded by the respondents as deeply meaningful: when asked how they felt about having had their encounter, 71.1% reported that they ‘treasured it’ and a further 20.5% were very glad it had happened. A large majority (73.4%) believed that the experience had brought them comfort and emotional healing, and 68.4% considered it to be important for their bereavement process. This pattern confirms Rees's original findings and the results of a number of conceptual replications.^11,38,39^ Some researchers have claimed that the ADC can be a sad, unpleasant or distressing experience, serving as a reminder of their loss and evoking feelings of loneliness.^3,4,23,40^ Nevertheless, only 11.9% reported that their perceived contact with a deceased loved one made their physical absence more painful. Many respondents in the current study (60.1%) reported that their fear of death had decreased or even disappeared, and (perhaps predictably) the proportion believing in life after death increased from 68.9% to 93.0%, which is consistent with Nowatzki and Kalischuk's^7^ finding that encounters profoundly affected the participants’ beliefs in an afterlife and attitudes toward life and death, and had a significant impact on their grief.

Although respondents typically describe their ADC as a religious/spiritual phenomenon,^41^ changes to religiosity and spirituality as a result of having an ADC has not been investigated previously. We asked respondents to estimate their degree of religiosity and spirituality before their experience and again after having had the experience. Although there were no changes in reported religiosity (*t*[983] = 0.371, *P* = 0.710, *d* = 0.02), levels of spirituality were significantly higher following an ADC (*t*[986] = 18.947, *P* < 0.0001, *d* = 0.60). These patterns are the same for male and female respondents (for religious change: *t*[975] = −1.666, *P* = 0.096, *d* = 0.14; for spiritual change: *t*[975] = 0.808, *P* = 0.419, *d* = 0.07). However, there were language group differences in effects upon religiosity (*F*[2, 980] = 6.755, *P* < 0.001, partial *η*^2^ = 0.014), with English and French respondents showing no change, but Spanish respondents showing a decrease after their ADC. For spirituality there were also language group differences (*F*[2, 983] = 11.732, *P* < 0.001, partial *η*^2^ = 0.023), with Spanish respondents showing the smallest increase and French respondents the largest increase.

Some forms of encounter were more impactful than others (see Table 3); tactile and olfactory ADCs produced significant shifts in religiosity, and tactile ADCs produced significant shifts in spirituality. Increases in spirituality were greater for those who reported that they had been in deep mourning at the time of the ADC (*t*[477] = 2.770, *P* = 0.006, *d* = 0.27), and who claimed to receive information that was previously unknown to them (*t*[894] = 1.904, *P* = 0.058, *d* = 0.15).

**Table 3:** Reported change in religiosity and spirituality by after-death communications type

		Religious change	*t*	*P-*value	Spiritual change	*t*	*P-*value
Sleep	Yes	−0.007	−0.427	0.669	0.568	0.173	0.863
	No	0.017			0.557		
Touch	Yes	0.066	2.509	0.012	0.657	2.619	0.009
	No	−0.061			0.493		
Sight	Yes	0.013	0.544	0.586	0.621	1.480	0.139
	No	−0.015			0.529		
Hearing	Yes	−0.026	−1.264	0.207	0.584	0.520	0.603
	No	0.039			0.552		
Sense of presence	Yes	−0.009	−0.200	0.842	0.524	−1.034	0.302
	No	0.002			0.591		
Smell	Yes	0.092	2.191	0.029	0.539	−0.344	0.731
	No	0.017			0.557		

## Discussion

### Reflection on study limitations and implications for practice

In this study, we revisited Rees's observation that many people experiencing bereavement have sensory encounters with their deceased loved one that are cherished by them and provide comfort as they gradually come to terms with their loss. By mapping the characteristic features and effects of ADCs among a relatively large sample, we sought to demystify these often-misunderstood experiences, and to explore how they can facilitate adaptive outcomes of grief. A limitation of the current design is that the veracity of accounts reported here cannot be independently verified, given that they are volunteered retrospectively and have been gathered by a method that does not allow for follow-up; we note, however, that in studies of ADCs that allowed respondents’ accounts to be tested by interviewing witnesses and checking official records, the incidence of distortion or outright deception is extremely low.^17,36^ Despite encouraging a broader range of respondents by providing three different language versions of the questionnaire, it seems likely that respondents were primarily from developed, capitalist, industrial countries that may broadly share cultural expectations about the possibility and form of ADCs. It would be valuable to extend this survey to other communities that do not share these expectations to see if this affected the phenomenology they report.

Our cases are consistent with the claim that ADCs often occur unexpectedly, and their likelihood seems independent of any underlying pathology or psychological need. Whatever their ontological status, they are perceived as ‘real’ by a great number of persons, and this orientation has tangible effects on them. Findings of this study support the emerging model of grief that posits that maintaining bonds with the deceased can be adaptive in circumstances where the person experiencing the ADC can make sense of their experience within culturally sanctioned (spiritual) conceptual frameworks.^10,29^ ADCs are typically regarded as deeply meaningful experiences that have enduring consequences. Clients or patients who might disclose them during bereavement counselling, could derive much benefit from the experience if they are afforded the opportunity to reflect and make sense of them in terms of their own beliefs and their historical relationship with the deceased. The high incidence of ADCs argues against any automatic attribution of dysfunction or pathology,^42^ and accepting an ADC as psychologically real can create a safe space for reflection without necessarily seeming to endorse beliefs about the experience that the therapist does not share. In contrast, attempts to trivialise or set aside experiences can be frustrating and distressing for the patient in ways that can become dysfunctional.^18^

## Data Availability

The data that support the findings of this study are available from the corresponding author, C.A.R., on reasonable request. A de-identified data-set will be made available at https://open-data.spr.ac.uk/ after a 24-month moratorium agreed with the funder of this project to allow findings to be published.

## Supplementary material

For supplementary material accompanying this paper visit http://dx.doi.org/10.1192/bjo.2021.960. click here to view supplementary material
