# Supplementary material for: The phenomenology and impact of hallucinations concerning the deceased
Source: BJPsych Open. 2021 Aug 16;7(5):e148. doi: 10.1192/bjo.2021.960 (PMC8388006; doi:10.1192/bjo.2021.960)
Supplement: Supplementary file 1 [file S2056472421009601sup001.pdf]

Questionnaire Introduction

Objective of research project

A perceived spontaneous and direct After-Death Communication (ADC) occurs when a mourner unexpectedly perceives a deceased person through the senses of sight, hearing, smell, or touch. Very commonly, persons who experience an ADC (experients) solely "feel the presence" of the deceased person or perceive a contact or a communication during sleep or hypnagogic states.

ADCs occur frequently, with research having found that 50% of mourners have experienced one or more spontaneous and direct ADCs. Testimonies collected in different countries and since the last century suggest this phenomenon to be universal and timeless. Despite their widespread occurrence, ADCs, paradoxically, have been little researched and are absent from the media and public discourse. As a consequence experients usually have no frame of reference in terms of which to understand, integrate and fully benefit from this experience which doesn't match mainstream conceptions of reality.

The objective of this international 2-year research project is to gain a better understanding of the phenomenology and the impact of perceived spontaneous After-Death Communications. The data collected on the basis of the present questionnaire are expected to provide insights into the profile of the experients; the profile of the deceased person allegedly initiating the contact; the circumstances of occurrence; the type, unfolding and message of ADCs; and their impact on experients. The outcome should permit disclosure of this hidden social phenomenon to the scientific community and the public by means of publications, conferences, and media events.

The project team thanks you very much for taking the time to complete this questionnaire. You can fill it in in several goes by using the save function which will allow you to resume the questionnaire later. Should you have any questions concerning the survey, please contact Dr Cal Cooper of the University of Northampton at callum.cooper@northampton.ac.uk

Consent: The following survey is exploring the experience and impact of After-Death Communications during bereavement. Taking part is at your own discretion and you are free to decline to answer any questions which may be too personal or distressing to share. We hope that you find this a positive experience, but if subsequently you change your mind and would rather withdraw from the study then you are free to do so up to 7 days after returning this questionnaire. If you wish to withdraw, please email Evelyn Elsaesser at evelyn@evelyn-elsaesser.com and provide your personal identifier which you will create on the next page.You do not have to give a reason. All information is given anonymously and no data are collected that could identify you personally. Please note that during the process of data analysis, all the names of persons and places will be anonymised. Please also consider the issues involved in this survey, particularly if matters of death and bereavement are a sensitive issue, before agreeing to take part. Please tick to confirm:

Please select exactly 7 answer(s).

☐ I have read and understood the Participant Information Page

☐ I have been given the contact details of the researcher and understand I can ask questions about the study if anything is unclear to me

☐ I understand that my participation is voluntary and that I am free to withdraw at any time during the survey and up to 7 days after completing it, without giving a reason. In such a case, all my data will be destroyed.

☐ I understand that the survey includes questions regarding death and bereavement and about unusual and personal experiences I have had concerning the deceased. I am free to omit any questions that I would rather not answer.

☐ I am aware that the outcomes of this study may be made public in talks and publications, but that the data will be a summary across all participants so that I will not be identifiable

☐ I understand and consent to the researcher holding any data collected in accordance with University guidelines and understand that my data will be held anonymously.

☐ I consent to my data being stored, archived, shared and re-used for future research, but I understand that I will still remain anonymous and unidentifiable from the data.

Contact Details

Q2: Personal identifier (your name or any information of your choice, e.g. birth date, nickname, etc.)

Confidentiality

Q3: Do you grant us permission to: -

- Use your testimony for research purposes and, where appropriate, also as illustration for the writing of books, articles, for conferences, etc.?
- Share your testimony with other researchers? Required

○ Yes

○ No

Personal Data

Q4: What is your gender?

- Male
- Female
- Other (e.g. transgender)

Q5: What is your age?

Q6: What is your marital status?

- Married
- Registered partnership (same-sex partnership)
- Single
- Separated
- Divorced
- Widowed

Q7: Do you have children?

- Yes
- Currently Pregnant
- No
- Other (yes, but deceased)

Q8: What is the highest level of education you have completed?

- University
- college or equivalent
- Intermediate between secondary level and university (e.g. technical training)
- Secondary school

Q9: Which of the following statements about occupational status apply to you?

- Part-time or hourly work
- Full-time work
- Looking for work
- In school
- In university
- In retraining
- On temporary leave (education leave, public service leave, etc.)
- In military/community/voluntary social service
- Exclusively house wife/ house man
- In age-related retirement
- In early retirement
- None of the above apply

Q10: Religious affiliation at the time of perceived ADC:

Q11: Before experiencing the perceived ADC, did you consider yourself a religious person?

- Strongly agree
- Agree
- Neutral
- Disagree
- Strongly disagree

Q12: Today, do you consider yourself a religious person?

- Strongly agree
- Agree
- Neutral
- Disagree
- Strongly disagree

Q13: Before experiencing the perceived ADC, did you consider yourself a spiritual person?

- Strongly agree
- Agree
- Neutral
- Disagree
- Strongly disagree

Q14: Today, do you consider yourself a spiritual person?

- Strongly agree
- Agree
- Neutral
- Disagree
- Strongly disagree

Multiple perceived ADCs

Q15: Did you experience several perceived ADCs?

- Yes
- No
- I'm not sure

Q16: If yes, how many?

Q17: Did you always perceive the same deceased person?

- Yes
- No
- I' m not sure

Q18: If you have experienced several perceived ADCs that seemed to involve the same deceased person, what was the interval between the different contacts? (minutes, hours, days, years)

Description of perceived ADC

Q19: Please describe in as much detail as you can your ADC. If you have experienced several ADCs with one or several deceased persons, please describe **only one** ADC, choosing the **most significant one**.

Additional Information by Type of Perceived ADC

In order to identify patterns across people's experiences, please answer the following questions that list some of the known types of perceived ADCs. The questionnaire will skip the questions that do not apply to your experience.

Q20: Did you perceive the **presence of the deceased**, ***without*** seeing, hearing, or feeling a physical contact of the deceased, or smelling a fragrance characteristic of the deceased? (if you saw, heard, felt a physical contact, or smelled a fragrance characteristic of the deceased please select 'No')

- Yes
- No
- I' m not sure

Q21: Could you, without seeing , hearing, or feeling the deceased, localise the presence in space? (you "knew" that the deceased was behind you, next to you, in front of you, etc.)

- Yes
- No
- I' m not sure

Q22: Did the impression of presence have a specific beginning and end?

- Yes, I knew exactly when the deceased came and left
- No, I didn't know exactly when the deceased came and left
- I'm not sure

Q23: Was the feeling of the deceased's presence the same or different from the times when you thought about the deceased and felt that he/she was "by your side" or " in your heart"?

- The same
- Different
- I'm not sure

Q24: If different, please explain

Q25: Did you feel that the deceased tried to communicate something by his / her sheer presence?

- Yes
- No
- I'm not sure

Q26: If 'Yes', please specify:

Audible Anomalies

Q27: Did you hear the deceased?

- Yes
- No
- I'm not sure

Q28: Did the voice come to you from the outside, as in a conversation between living persons?

- Yes
- No
- I'm not sure

Q29: Was the voice the same or different from the one the deceased had while alive?

- The same
- Different
- I'm not sure

Q30: If different, please describe

Sound or Telepathy

Q31: Was the communication without an external sound, as if perceived telepathically?

- Yes
- No
- I'm not sure

Q32: Was the perceived communication different from a thought?

- Yes
- No
- I'm not sure

Physical Contact

Q33: Did you ***feel*** a physical contact initiated by the deceased? (e.g. you felt that the deceased was touching your hand, wrapping their arm around your shoulders, etc.)

- Yes
- No
- I'm not sure

Q34: In what part of your body did you feel the contact and how did it occur?

Q35: Was this contact familiar to you / was it typical for the deceased?

- Yes
- No
- I'm not sure

Q36: Did you feel that this contact was transmitting some sort of message?

- Yes
- No
- I'm not sure

Q37: If yes, please specify

Q38: What did this contact feel like?

Visual Anomalies

Q39: Did you *see* the deceased?

- Yes
- No
- I'm not sure

Q40: When you saw the deceased, were your eyes:

- Open
- Closed (you saw the deceased in your mind)
- I'm not sure

Q41: Did you perceive the deceased as a whole or only a part of the body?

- As a whole
- Only upper part of body
- Only lower part of body
- I'm not sure
- Other

Q42: If other, please explain:

Q43: Were the feet visible?

- Yes
- No
- I'm not sure

Q44: Was the deceased positioned

- In the centre of my vision (in front of me)
- In the periphery of my vision (at my side)
- I'm not sure

Q45: At what distance did you perceive the deceased?

- Within reaching distance
- Several metres away
- In the distance
- I'm not sure

Q46: What was the consistency of the deceased?

- seemed solid like a living being
- semi-transparent (objects behind apparition were visible)
- a foggy silhouette
- Other

Q47: If *other*, please explain:

Q48: Did the deceased look the same as they did at the hour of their death (visibly marked by their illness, by the accident that caused their death, etc.) or didn't they look the same?

- Yes, looked the same
- No, looked different
- I'm not sure
- Other

Q49: If other, please explain:

Q50: Was the deceased immediately familiar to you, e.g. wearing their favourite cloths, behaving in a familiar, way, etc.

- Yes
- No
- I'm not sure

Q51: Did the deceased seem to be the same age as when he/she died?

- Same age
- Younger
- Older
- I'm not sure

Q52: Did the deceased seem brighter than their environment, e.g. was there a light that surrounded them or emanated from them?

- Yes
- No
- I'm not sure

Q53: How did the deceased appear?

- Materializing right before my eyes
- Already there when I perceived them
- I'm not sure

Q54: Was the deceased

- Completely motionless
- Quite static
- Moving about the environment
- Quite animated (e.g. gesticulating)
- I'm not sure

Q55: How did the apparition disappear?

- Fading gradually
- Dissolving instantly
- Not there anymore when I blinked
- I'm not sure
- Other

Q56: If "other", please explain

Q57: Did you feel that this apparition was conveying a message to you?

- Yes
- No
- I'm not sure

Q58: If yes, was the message conveyed to you:

- In words you could hear (like a conversation among living persons)
- As if telepathically
- By the expression of the face
- I'm not sure
- Other

Q59: If 'yes', please specify

Q60: If other, please explain

Touching the Deceased

Q61: Did you try to touch the deceased?

- Yes
- No
- I'm not sure

Q62: If yes, could you grasp the apparition/did you feel a resistance/matter?

- Yes
- No
- I'm not sure

Q63: Please explain your answer in more detail:

Smell Anomalies

Q64: Did you smell a fragrance characteristic of the deceased which made you think he/she was present?

- Yes
- No
- I'm not sure

Q65: If yes, describe the fragrance:

Q66: Did you feel that the deceased was conveying a message to you by way of this fragrance?

- Yes
- No
- I'm not sure

Q67: If 'yes', please specify

Sleep Anomalies

Q68: Do you feel that you have been contacted by the deceased **during your sleep**?

- Yes
- No
- I’m not sure

Q69: Were you soundly asleep or just falling asleep or just waking up?

- Soundly asleep
- Falling asleep
- Waking up
- I’m not sure

Q70: Was it a dream?

- Yes
- No
- I’m not sure

Q71: If no or uncertain, how was this experience different from a dream?

At the Moment of Death

Q72: Did you experience a perceived ADC at the moment of death, in other words, the dying/ deceased came to you in some form of ADC to tell you they were dying/dead?

- Yes
- No
- I’m not sure

Q73: Was the moment (hour/minute) when you experienced the perceived ADC later confirmed as being the actual hour of death of the person?

- Yes
- No
- I’m not sure

Q74: Was the demise predictable or expected (the person was very ill, very elderly, undergoing life-threatening surgery, etc.)?

- Yes
- No
- I’m not sure

Q75: Were you at a distant location?

- Yes
- No
- I’m not sure

Q76: Did you have the opportunity to say good-bye or to resolve unfinished business before they died?

- Yes
- No
- I’m not sure

Additional Information for all Types of Perceived ADCs

Q77: Did you immediately and beyond doubt recognize the identity of the deceased?

- Yes
- No
- I'm not sure

Q78: How long did the contact last?

- Some seconds
- Some minutes
- Longer
- I'm not sure

Q79: Did the experience happen instantly or gradually?

- Instantly
- Gradually
- I'm not sure

Q80: Did you perceive the emotional state / mood of the deceased

- Yes
- No
- I'm not sure

Q81: If yes, in what emotional state / mood was the deceased? (please check all proposals that apply)

- Calm
- Radiant with bliss
- Eager to comfort
- Compassionate
- Sad
- Agitated
- Worried for you
- Frightened
- Threatening
- In another state of mind

Q82: If *in another state* of mind, please explain:

Q83: When you perceived the contact seemingly created by the deceased, were you:

- Alone
- With another person
- With several persons
- I'm not sure

Q84: If you were with other person(s) during the perceived ADC

- I was the only one to perceive the contact
- The other person(s) also perceived the contact
- I'm not sure

Q85: If the other person(s) present also perceived the contact, please describe what they perceived

Q86: Did another person(s) you know have a contact with the same deceased person at some point?

- Yes
- No
- I'm not sure

Q87: If yes, please explain

Q88: Was a pet (cat, dog, etc.) in the same place at the time of the perceived ADC?

- Yes
- No
- I'm not sure

Q89: If yes, did you notice that the pet was behaving in an unusual way?

- Yes
- No
- I'm not sure

Q90: If yes, please describe the behaviour of the pet

Message Perceived

Q91: Did you receive a message from the deceased during the perceived ADC?

- Yes
- No
- I'm not sure

Q92: If yes, please describe the message received

Q93: Did you receive any information unknown to you previously?

- Yes
- No
- I'm not sure

Q94: If yes, please describe previously unknown information

Q95: How was the message conveyed by the deceased

- By words that were audible, such as an external voice
- As if telepathically (the message was "put in your mind")
- By facial expression (for visual perceived ADCs)
- I'm not sure
- Other

Q96: If other, please explain

Circumstances of Perceived ADC

Q97: How much time has elapsed since you had the experience?

Q98: How much time had elapsed between death and perceived ADC (in hours, days, months or years):

Q99: Did you know that the person was deceased at the moment of the perceived ADC?

- Yes
- No
- I'm not sure

Q100: Where did the experience occur? (e.g. at home, in the garden, in the car, at work, in the city, in nature, etc.)

Q101: At what time of the day / night did the perceived ADC occur?

- In the morning
- In the afternoon
- In the evening
- During the night
- I'm not sure

Q102: Was the place where the perceived ADC occurred:

- Illuminated by the light of day
- Illuminated by an electric light
- Dimly lit by twilight, an outside light, etc.
- In the dark
- I'm not sure
- Other

Q103: If other, please explain

Q104: In what state of alertness were you at the time of the perceived ADC?

- Completely awake and active
- Completely awake and resting
- Somnolent / half asleep
- Asleep
- Falling asleep/Waking up (hypnagogic state)
- I'm not sure
- Other

Q105: If other, please explain

Q106: If you were asleep, did the perceived ADC wake you up or did it occur while you were sleeping?

- The perceived ADC woke me up
- The perceived ACD occurred while I was sleeping
- I'm not sure

Q107: What were you doing at the moment the perceived ADC occurred?

Frightening Perceived ADC

Q108: Were you frightened by the contact seemingly created by the deceased?

- Yes
- No
- I'm not sure

Q109: If yes, were you frightened

- During the whole perceived ADC
- At the beginning only
- At the end only

Q110: Were you frightened because

- I was destabilized by the fact that the deceased was apparently able to establish a contact with me
- I feared to be losing my mind/hallucinating
- I thought the deceased's intention was to harm me
- other

Q111: If other, please explain

Q112: Did the contact immediately stop when you were frightened?

- Yes
- No
- I'm not sure

Q113: If *yes*, how did you feel about the interrupted contact?

- I was relieved
- I regretted
- I didn't mind
- I'm not sure

Partial Temporary Paralysis

Q114: Did you at any time during the perceived contact with the deceased notice some sort of a partial temporary paralysis?

- Yes
- No
- I'm not sure

Q115: Did you feel that you could not **move** during the alleged contact with the deceased?

- Yes
- No
- I'm not sure

Q116: Did you feel that you could not **speak** during the alleged contact with the deceased?

- Yes
- No
- I'm not sure

Q117: Did you notice some other sort of physical restriction/partial temporary paralysis?

- Yes
- No
- I'm not sure

Q118: If yes or uncertain, please explain:

Q119: Was this partial temporary paralysis frightening?

- Yes
- No
- I'm not sure

Q120: Did the contact stop when you were frightened?

- Yes
- No
- I'm not sure

Q121: Please feel free to add additional comments on this partial transient paralysis

Emotional State at the Moment of Perceived ADC

Q122: What was your state of health at the moment of the perceived ADC?

- Good health
- Sick
- Depressed
- Under medication (anti-depressant, etc.)
- Under the influence of substances (recreational drugs, alcohol, etc.)
- I'm not sure

Q123: In your own words, please describe your emotional state immediately before the perceived ADC:

Q124: Did you think about the person since his/her death

- Almost constantly
- Several times per day
- Often
- Sometimes
- Rarely
- Very rarely
- Never
- I'm not sure
- Deceased was unknown to me

Q125: In the minutes preceding the perceive ADC, were you thinking about the deceased?

- Yes
- No
- I'm not sure

Q126: During the days / weeks previous to the perceived ADC, and in terms of the bereavement process, were you (please check the answer that best fits):

- Extremely sad and in deep mourning
- Moderately sad and moderately mourning
- A little sad but having already overcome the pain of mourning
- Not sad and not mourning any more
- I have never been in mourning (of the person perceived)
- I'm not sure

Emotions Associated with Perceived ADC

Q127: Describe what you felt during the perceived ADC

Q128: Describe what you felt immediately after the perceived ADC

Q129: Describe what you feel now while remembering the perceived ADC

Q130: How do you feel about having had this experience?

- I treasure it
- Very glad
- I don't mind
- Very unhappy
- I wish it had never happened
- I'm not sure

Belief System After Perceived ADC

Q131: Did you believe in life after death ***before*** your perceived ADC?

- Yes
- No
- I'm not sure

Q132: ***Today***, do you believe in life after death?

- Yes
- No
- I'm not sure

Q133: Did you believe that deceased persons can contact living persons ***before*** your perceived ADC?

- Yes
- No
- I'm not sure

Q134: ***Today,*** do you believe that deceased persons can contact living persons?

- Yes
- No
- I'm not sure

Q135: Did you notice a change in your perception of death following the perceived ADC

- Yes
- No
- I'm not sure

Q136: If yes, please explain

Q137: Following the perceived ADC, did your fear of death

- Decrease
- Disappear
- Remain the same
- Increase
- I'm not sure

Q138: Do you consider the perceived ADC to be

- Life-changing
- Important
- Moderately important
- Not very important
- Not important
- I'm not sure

Q139: Did you share your perceived ADC with family members, friends, or other persons?

- Yes
- No
- I'm not sure

Q140: If yes, please explain with whom you shared your experience and how it was received

Q141: If no, please explain why you didn't share your experience with anybody

Q142: Has your understanding of life and death changed after the perceived ADC?

Q143: Did you ever consult a spirit medium with the intention to establish a contact with a deceased person before your perceived ADC?

- Yes
- No
- I'm not sure

Q144: If yes, how often?

Q145: Did you ever consult a medium (channel) with the intention to establish a contact with a deceased person after your perceived ADC?

- Yes
- No
- I'm not sure

Q146: If yes, how often?

Information on the Perceived Deceased

Q147: Was it a man, a woman, or a child (0-16 years)?

- A man
- A woman
- A child
- Other

Q148: If other, please explain

Q49: Who was the deceased in relation to you? (e.g., grandmother, friend, acquaintance, unknown etc.)?

Q150: How old was the person when he/she died?

Q151: What was the cause of death?

- Disease
- Cardiac arrest
- Accident
- Old age
- Suicide
- Accident
- Natural disaster
- Murder
- I'm not sure
- Other

Q152: If other, please explain

Q153: If the person was ill, did he/she die

- Suddenly
- After a short illness
- After a long illness
- I'm not sure

Q154: Did the person die

- In hospital
- At home
- At the scene of the accident/heart failure, etc.
- I'm not sure
- Other

Q155: If other, please explain

Q156: Was the deceased a

- Strong believer (in religious terms)
- Moderate believer (in religious terms)
- Spiritual (outside any specific religious institution)
- Agnostic
- Atheist
- I am not sure

Q157: Did the deceased believe in life after death?

- Yes
- No
- I'm not sure

Relationship with Deceased

Q158: Did you know the deceased person who seemingly created the ADC?

- Yes
- No
- I'm not sure

Q159: What was your emotional connection with the deceased

- Extremely close and loving
- Very close
- Quite close
- Quite distant
- Distant
- Confrontational
- Extremely difficult
- Deceased was unknown to me
- I'm not sure

Q160: Did you and the deceased have serious unresolved conflicts ("Unfinished business")?

- Yes
- No
- I'm not sure

Q161: If yes, describe unresolved conflict

Q162: Had you and the subsequently deceased person ever discussed the possibility of contacts between the living and the deceased?

- Yes
- No
- I'm not sure

Q163: Had you and the subsequently deceased person an agreement saying "the first who dies tries to contact the other?

- Yes
- No
- I'm not sure

Impression of Reality of the Perceived ADC

Q164: Before your experience, had you ever heard of perceived ADCs?

- Yes
- No
- I'm not sure

Q165: If you had heard of perceived ADCs before your own experience, did you think they: (Please check the answer that fits best)

- Were most certainly authentic
- Open to the idea, but needed evidence
- It was unlikely that they would be authentic
- Were certainly not authentic (but rather an hallucination/illusion created through grief)
- I did not ask myself the question
- I was not sure

Q166: Do you think that your prior knowledge of perceived ADCs has influenced your own experience?

- Yes
- No
- I'm not sure

Q167: If yes or uncertain, how do you think it influenced your own experience?

Q168: Shortly after your experience (a few hours/days after the perceived contact), did you think that the perceived ADC: (Please check the answer that fits best)

- Was authentic beyond doubt
- Could possibly really have happened
- It was unlikely that it was authentic
- Was certainly not authentic but rather a hallucination/illusion created by grief
- I did not ask myself the question
- I was not sure

Q169: Today, with hindsight, do you think that your experience: (please check answer that fits best)

- Was authentic beyond doubt
- Could possibly really have happened
- It is unlikely that it was authentic
- Was certainly not authentic but rather a hallucination/illusion created by grief of bereavement
- I'm not sure

Bereavement Process

Q170: Has the perceived ADC brought you comfort / emotional healing?

- Yes
- No
- I'm not sure
- Not applicable (I was never mourning this person)

Q171: If yes, please explain

Q172: Does having had a perceived contact with the deceased make his/her physical absence even more painful?

- Yes
- No
- I'm not sure

Q173: Do you consider the perceived ADC important for your bereavement process?

- Yes
- No
- I'm not sure

Q174: If yes, or uncertain please specify

Q175: How would you describe your relationship with the deceased?

- My relationship ended when he/she died
- I thought that the relationship ended with death but my perceived ADC revealed that the bond continues
- I believed that my bond with the deceased continued after death and my perceived ADC deepened the connection
- I'm not sure
- Other

Q176: If other, please explain

Q177: Do you really wish for more contact with the deceased or is/are the contact(s) perceived already sufficient for you?

- I really wish for a new contact
- The perceived contact(s) is/are sufficient for me
- I'm not sure
- Other

Q178: If other, please explain

Q179: The grieving process is characterized by several stages, including shock, denial and anger. Do you feel that your perceived ADC has had an influence on any of these?

- Yes
- No
- I'm not sure

Q180: If yes, please explain

Q181: Did the perceived ADC affect the sadness triggered by the loss of the person or did the sadness remain the same?

- Sadness is reduced
- Sadness is removed
- Sadness remains the same
- Sadness is increased
- I'm not sure
- Other

Q182: If other, please explain

Q183: Has the perceived ADC made it easier to accept your loss?

- Yes
- No
- I'm not sure

Q184: Do you think that your recovery and return of the taste for life have been accelerated by the perceived ADC?

- Yes
- No
- I'm not sure

Q185: Do you believe that the bereavement process would have been the same or different if you had not experienced the perceived ADC?

- The same
- Different
- I'm not sure

Q186: Please explain your choice:

Near-Death Experiences, OBEs, and Other Types of Spiritual Experiences

Q187: Did you ever have a Near-Death Experience (NDE)? (i.e. a perceived experience while dying and then revived)

- Yes
- No
- I'm not sure

Q188: If yes, please describe your experience briefly (date, circumstances, and unfolding)

Q189: Did you ever have a spontaneous Out-of Body Experience (OBE)? (i.e. a feeling of being literally out of your own body, and able to travel freely with no physical form)

- Yes
- No
- I'm not sure

Q190: If yes, please describe your experience (date, circumstances, and unfolding)

Q191: Did you ever have any other kind of spontaneous spiritual experience?

- Yes
- No
- I'm not sure

Q192: If yes please describe your experience (date, circumstances, and unfolding)

Q193: Did you ever have a spontaneous psychic experience (e.g. telepathy, etc.)?

- Yes
- No
- I'm not sure

Q194: If yes please describe your experience (date, circumstances, and unfolding)

